# An annotated water-filled, and dry potholes dataset for deep learning applications

**DOI:** 10.1016/j.dib.2023.109206

**Published:** 2023-05-06

**Authors:** Jihad Dib, Konstantinos Sirlantzis, Gareth Howells

**Affiliations:** a School of Engineering, University of Kent, Canterbury, Kent CT2 7NZ, United Kingdom; b School of Engineering, Technology and Design, Canterbury Christ Church University (CCCU), North Holmes Road, Canterbury, Kent, CT1 1QU, United Kingdom

**Keywords:** Deep learning, Transfer learning, Artificial neural networks, Potholes, Images Dataset, Water-Filled Potholes, Dry Potholes

## Abstract

Potholes have long posed a challenging risk to automated systems due to their random and stochastic shapes and the reflectiveness of their surface when filled with water, whether it is “muddy” water or clear water. This has formed a significant limitation to autonomous assistive technologies such as Electric-Powered Wheelchairs (EPWs), mobility scooters, etc. due to the risk potholes pose on the user's well-being as it could cause severe falls and injuries as well as neck and back problems. Current research proved that Deep Leaning technologies are one of the most relevant solutions used to detect potholes due to the high accuracy of the detection. One of the main limitations to the datasets currently made available is the lack of photos describing water-filled, rabble-filled, and random coloured potholes. The purpose of our dataset is to provide the answer to this problem as it contains 713 high-quality photos representing 1152 manuall-annotated potholes in different shapes, locations, colours, and conditions, all of which were manually-collected via a mobile phone and within different areas in the United Kingdom along with two additional benchmarking videos recorded via a dashcam.

**Specifications Table**

**Table:** 

Subject	Computer science: Artificial Intelligence
Specific subject area	Pattern Recognition, Object Localisation, Image Understanding, Computer Vision, Deep learning, Object detection, Image processing, Convolutional Neural Networks
Type of data	Images YOLO TXT Format Annotations Pascal VOC XML Format Annotations
How the data were acquired	Images were acquired via the use of a normal 13 Megapixels phone camera Annotations were made via the use of a labelling tool and exported to Darknet Format
Data format	Raw JPG TXT Annotation XML Annotation
Description of data collection	Images were collected within different areas and different times of the day, and in different light conditions. Pothole images have been captured from different angles representing different shapes and conditions (water-filled, or dry).
Data source location	Institution: School of Engineering and Digital Arts, University of Kent City/Town/Region: Canterbury, Kent Country: United Kingdom
Data accessibility	Repository name: Mendeley Data Data identification number: Dib, Jihad; Sirlantzis, Konstantinos; Howells, Gareth (2023), “An Annotated Water-Filled, and Dry Potholes Dataset for Deep Learning Applications”, Mendeley Data, V1, doi: 10.17632/tp95cdvgm8.1 Direct URL to data: https://data.mendeley.com/datasets/tp95cdvgm8
Related research article	Dib, J., Sirlantzis, K. and Howells, G. (2022) ‘Application of Deep Learning Techniques in Negative Road Anomalies Detection’, in. 14th International Joint Conference on Computational Intelligence - ROBOVIS, pp. 475–482. doi:10.5220/0,011,336,000,003,332 (https://www.scitepress.org/PublicationsDetail.aspx?ID=cOZefN+26lU=&t=1)

## Value of the Data

• The different conditions of the potholes represented in this dataset are essential for any AI-based system as they provide sufficient information to cover most examples of potholes irrespective of the random stochastic shapes, light conditions, and general conditions (water-filled or dry).
• The data provided is essential to researchers in computer vision, robotics, autonomous vehicles, and road safety.
• This dataset is the first dataset that focuses on both water-filled and dry potholes.
• Data can be reused for different research purposes varying from autonomous vehicles/platforms to driverless cars, vehicle path planning, autonomous pothole detection and reporting for local authorities. Moreover, this data could provide an answer to the problems and limitations discussed in [1].

## 1 Objective

Our dataset has been generated as a solution to the lack of diversity in the widely-available pothole datasets. We are providing different photos describing water-filled, rabble-filled, and differently-shaped potholes. We are also providing sufficient amount of information in the form of surrounding pixels describing the background of the image around the pothole in order to provide the deep learning networks with enough information to detect the object of interest. This would enable researchers to overcome the limitation posed to assistive technologies and/or autonomous vehicles. In our research, this dataset enabled us to train and benchmark a neural network to detect and localise potholes in real-time with a very high accuracy and detection rate.

## 2 Data Description

The proposed dataset has been manually collected in order to provide an accurate solution to the current limitation of pothole detection systems, that is potholes, which are filled with water, ice, debris, and other factors limiting the accuracy of detection systems. There are some publicly available datasets; however, they do not cover potholes at night, clear and reflective water-filled potholes, and potholes filled with debris.

**Fig. 1:**
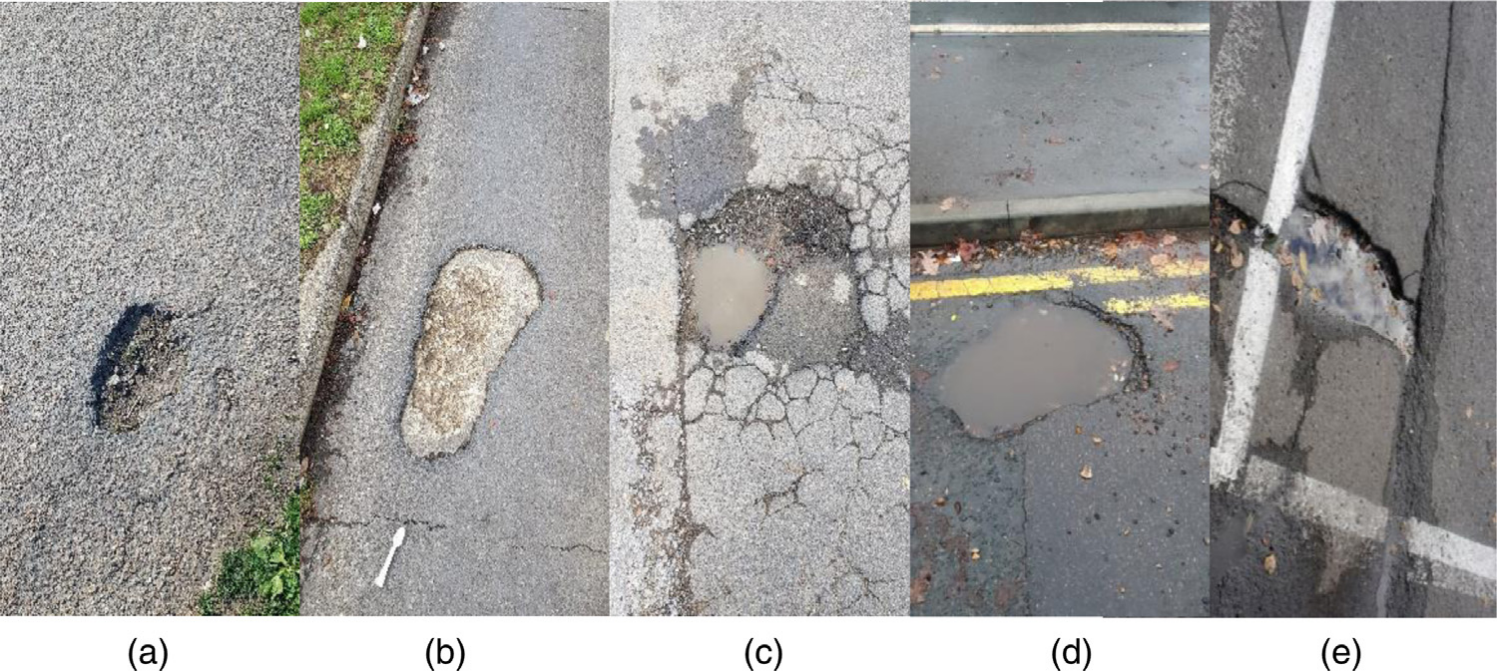
Samples images taken from our manually-collected dataset.

Fig. 1 represents a small example of the images collected within the dataset.

(a) A general pothole represented as a semi-circular hole within the tarmac.
(b) An almost depthless cracked surface within the tarmac, these types of potholes are usually a limitation to laser-based detection systems.
(c) A randomly-shaped pothole filled with water, and dirt, and some rabble on the side. This poses a limitation to laser, lidar, and sonar-based systems.
(d) A stochastic-shaped pothole filled with water that is almost clear, this pothole is located near the yellow-lines on the side of the road. This poses a limitation, and a challenge to the widely-used systems relying on image processing, laser, sonar, or lidar-based detection principles.
(e) A random-shaped crack on the side of the road filled with water, rabble, and leaves, and located on the side of a marked parking bay. This type of potholes is a difficult challenge to systems based on image processing, laser detection, LIDAR, and sonar detection systems due to the reflectiveness of the water and the randomness in the way the rabble is distributed.

**Fig. 2:**
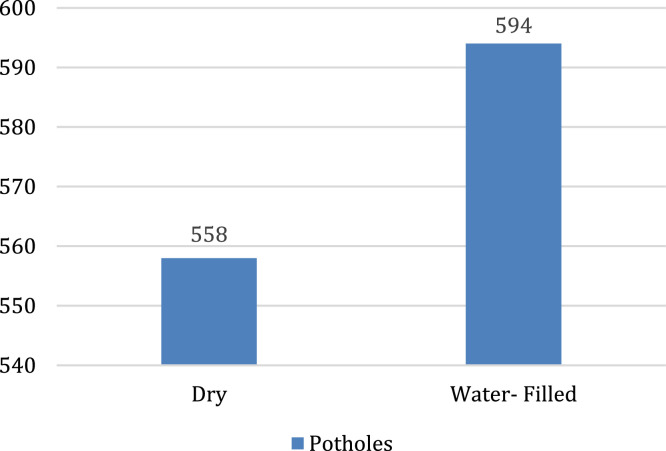
Distribution of potholes in the proposed dataset.

The proposed dataset is comprised of 713 labelled manually-labelled images containing 1157 potholes, distributed as per Fig. 2.

It can be noticed from Fig. 2 that the water-filled potholes dominate in the proposed dataset. This is due to the lack of clear, reflective, and muddy water-filled pothole images in the datasets publicly available.

**Fig. 3:**
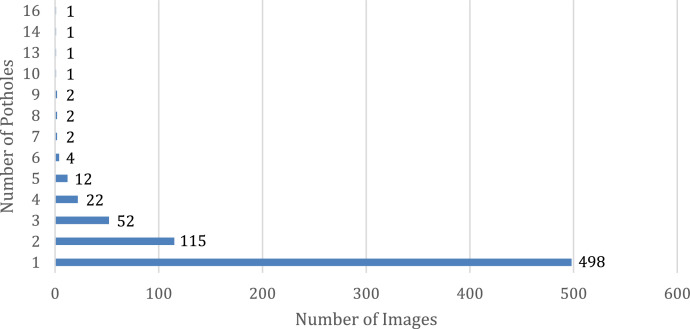
Number of Potholes in Images.

Fig. 3 shows the distribution of the number of potholes per image within the dataset.

It can be noticed that most of the images within the dataset contain one pothole, with the remaining photos containing two or more potholes.

After calculating the ratio of the area of every bounding box over the area of every image, we can obtain the average ratio of the area of potholes as per the formula below:

$$Ratio=\sum \frac{Height_{\;\left(Bounding\;Box\right)}\;\times \;Width_{\;\left(Bounding\;Box\right)}}{Height_{\;\left(Image\right)}\;\times \;Width_{\;\left(Image\right)}}\;=28.7529\%$$

This means that the images within the dataset contain enough information for any system to properly segment the pothole from the background after properly understanding the surrounding.

**Fig. 4:**
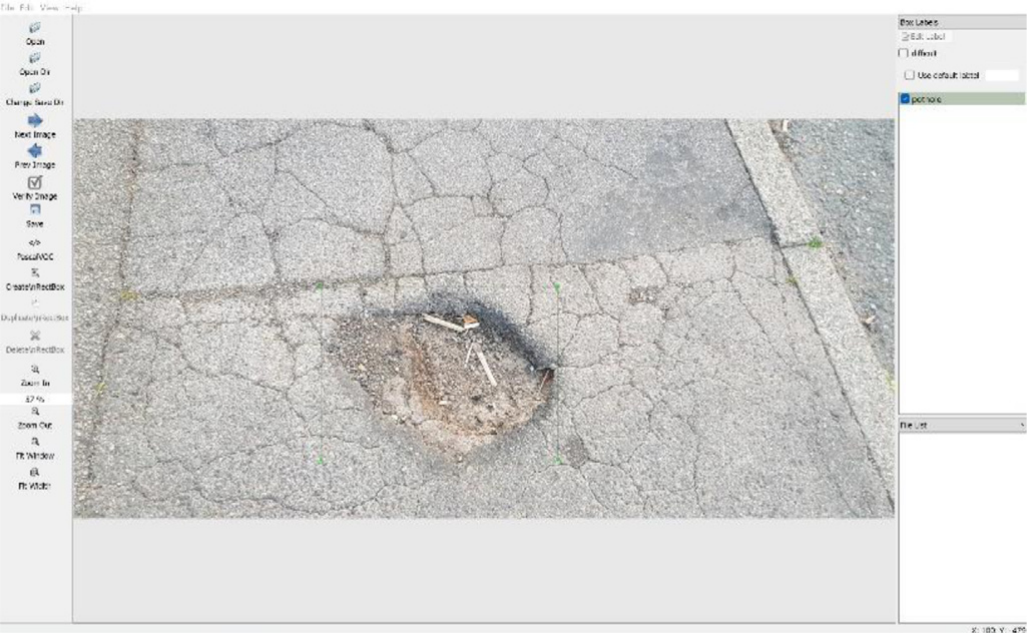
Labeling the dataset images via LabelImg showing how objects are labelled.

The collected images undertook a pre-processing stage where they were downscaled to 30% of the original size in an effort to obtain a width close to 415 pixels in order to ensure that our dataset is compatible the with most of the currentstate-of-the-art object detection algorithm. Then, images were individually labelled using LabelImg [2] for Python, an open-source labelling software. During the labelling process, all the bounding boxes were drawn taking into consideration that the corners of the pothole are exactly contained within the bounding box in order to minimise the number of pixels describing the background of the image as demonstrated in Fig. 4. This method helps in avoidingdivergence in the training process.

**Fig. 5:**
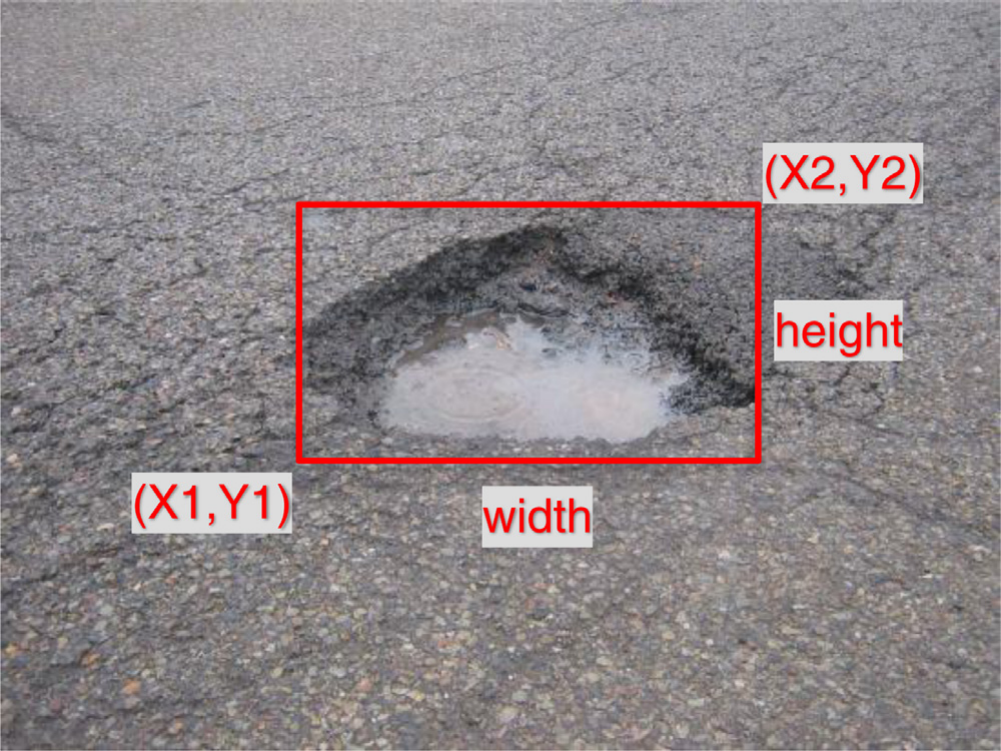
YOLO Darknet Annotation Format.

The annotations were saved in two different formats, YOLO Darknet [3], which is a text-based annotation where the .TXT, which has the same name as the image contains the class number (in this case, one class has been used, and it is “Pothole”), the X1,Y1 and X2,Y2 coordinates of the Object, along with the height, and width of the bounding box as per Fig. 5.

In addition to the YOLO Darknet format, images were annotated in the PASCAL VOC XML format, where the annotation file is an XML file that has the same name as the image, and contains the annotation data contained within an “<annotation>” tag where the name of the image file, its location, and size (width, height, and depth) are described. Then, the bounding box of the object is represented by the 〈bndbox〉 tag where xmin,ymin,xmax, and ymax are described, these are equivalent to x1,y1,x2,y2 of the YOLO Darknet format. Other tags such as truncated, segmented, and difficult are set to 0 as they do not apply in this case.

**Fig. 6:**
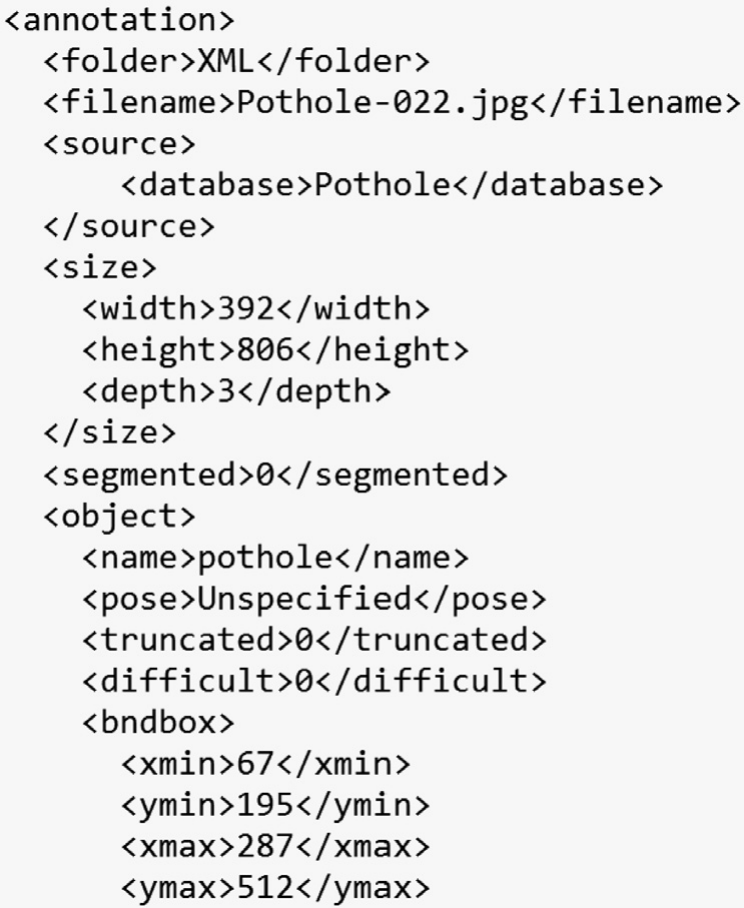
Pascal VOC XML Annotation Format.

Fig. 6 represents an example of one of the PASCAL VOC XML files.

The annotation formats chosen represent the widely used formats by the most popular object detection environments. This will enable researchers to easily incorporate the proposed dataset in their research and to train their object detection models swiftly and without the need to convert the annotation files to different formats.

## 3 Experimental Design, Materials and Methods

The images were collected using a Samsung Galaxy Note 8 13 Megapixels phone camera. Photos were taken within Kent County in the United Kingdom in different areas, cities, roads, and footpaths, and in various weather conditions (sunny, cloudy, and rainy days and nights).

In our research, some additional preprocessing techniques were used during the training process, such as data augmentation, bounding box regression, etc. However, the proposed dataset contains raw data without any preprocessing other than resizing the images to around 412×412 pixels when possible and ensuring that either the height or width is 412 pixels otherwise, as mentioned before. This enables future developers to flexibly and freely decide their application methods and to implement the preprocessing techniques which are best for their research-specific proposed methods.

As for the training and validation ratios, we have used an 80/20 ratio where 80% of the images were randomly used for training, and 20% were randomly used for validation. We recommend doing the same by randomising the split so that images are split after random shuffling as appose to hard-splitting them by simply splitting the images into two sets which are to be used for training and validation respectively.

The proposed dataset contains a significant number of pixels representing random backgrounds and objects. As shown in the previous section, the average ratio of the area of the pothole in comparison with the overall area of the image is 28.7529%. This enables the deep-learning, or AI-based algorithms which are to be used to accurately recognise potholes regardless of the time of the day, the weather, or the location of the pothole as they are provided with enough information about the background, and the surrounding enviorment.

## Ethics Statements

The authors generally followed the expected standards of ethical behaviour in scientific publishing throughout the article construction.

The work did not involve the use or participation of human subjects or animals.

## CRediT authorship contribution statement

**Jihad Dib:** Conceptualization, Methodology, Visualization, Formal analysis, Writing – original draft, Writing – review & editing, Data curation. **Konstantinos Sirlantzis:** Methodology, Validation, Resources, Supervision, Project administration, Writing – review & editing. **Gareth Howells:** Supervision, Methodology, Writing – review & editing.

## Declaration of Competing Interest

The authors declare that they have no known competing financial interests or personal relationships that could have appeared to influence the work reported in this paper.

## Data Availability

An Annotated Water-Filled, and Dry Potholes Dataset for Deep Learning Applications (Original data) (Mendeley Data).

## Appendix Supplementary materials

Image, application 1
