# Supplementary material for: An annotated water-filled, and dry potholes dataset for deep learning applications
Source: Data Brief. 2023 May 6;48:109206. doi: 10.1016/j.dib.2023.109206 (PMC10197008; doi:10.1016/j.dib.2023.109206)
Supplement: Supplementary file 1 [file mmc1.pdf]

Application of Deep Learning Techniques in Negative Road Anomalies Detection

Jihad Dib*[a], Konstantinos Sirlantzis[b] and Gareth Howells[c]
School of Engineering and Digital Arts, Jennison Building, University of Kent, Canterbury CT2 7NT, U.K.

Keywords: Pothole Detection, Road Anomaly, Deep Learning, Deep Neural Network, Convolutional Network, Image Processing, Object Detection, Object Classification.

Abstract: Negative Road Anomalies (Potholes, cracks, and other road anomalies) have long posed a risk for drivers driving on the road. In this paper, we apply deep learning techniques to implement a YOLO-based (You Only Look Once) network in order to detect and identify potholes in real-time providing a fast and accurate detection and sufficient time for proper safe navigation and avoidance of potholes. This system can be used in conjunction with any existing system and can be mounted to moving platforms such as autonomous vehicles. Our results show that the system is able to reach real-time processing (29.34 frames per second) with a high level of accuracy (mAP of 82.05%) and detection accuracy of 89.75% when mounted onto an Electric-Powered Wheelchair (EPW).

1 INTRODUCTION

Negative Road Anomalies is the term we have chosen to describe potholes, cracks, and any anomaly located at a negative position of the road surface.

Potholes pose the highest risk in all negative road anomalies as they are a danger to drivers when driving on roads, and im some cases motorways. They could cause severe injury to the driver in form of neck pain, back pain, whiplash and more severe health risks. Not to mention, the damage which could be caused to the car's mechanical system and tires putting the driver under numerous risks of accidents and even threatening their life as a result of a torn tire or other mechanical damage which could be caused to the vehicle driven when passing over a pothole at high speed. Potholes were also a significant limitation to the driverless car advancement projects due to the stochasticity of their nature and the difference in their depth and severity making them hard to identify and detect, and rendering many detection techniques futile as they sometimes contain some characteristics which could fall within the limitations of the detection techniques (for example, water-filled potholes cannot be detected via ultrasonic sensing techniques).

In our ongoing project, we apply deep learning techniques in order to detect and identify potholes. Our project is mainly focused on object detection neural networks which can be used in real-time in order to detect and classify potholes from the video stream obtained through the use of an RGB Camera, and to provide a fast and reliable detection method which allows sufficient time and distance for a safe avoidance and navigation of manual and autonomous vehicles and moving platforms.

YOLO (Redmon et al.,2015) (You Only Look Once) is the candidate network which is used in this project due to its high accuracy and fast performance, especially in real-time detection scenarios. The project was implemented over the Darknet (Redmon et al.,2020) environment which was developed by the authors and creators of YOLO and was optimized and tested in real-time scenarios where it returned significant promising results.

Many attempts to detect potholes were made, and different technology was used in order to implement solutions to the proposed problem. Some solutions were implemented via the use of laser imaging as

[a] https://orcid.org/0000-0002-3070-9673
[b] https://orcid.org/0000-0002-0847-8880
[c] https://orcid.org/0000-0001-5590-0880

Dib, J., Sirlantzis, K. and Howells, G.
Application of Deep Learning Techniques in Negative Road Anomalies Detection.
DOI: 10.5220/0011336000003332
In *Proceedings of the 14th International Joint Conference on Computational Intelligence (IJCCI 2022)*, pages 475-482
ISBN: 978-989-758-611-8; ISSN: 2184-2825
Copyright © 2022 by SCITEPRESS – Science and Technology Publications, Lda. All rights reserved

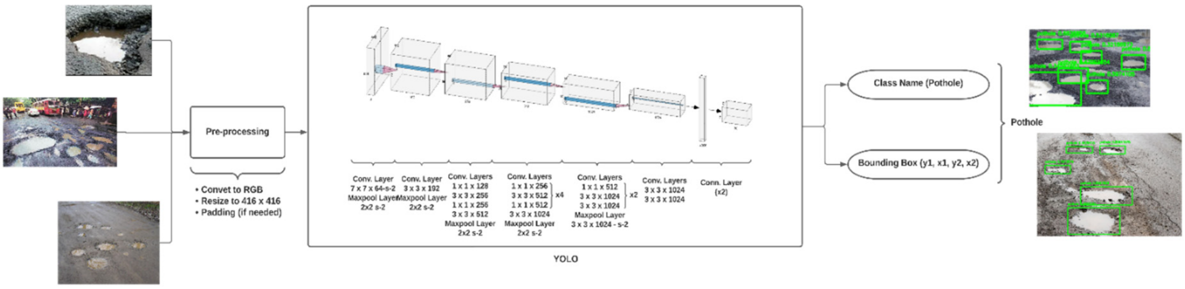

Figure 1: Pothole Detection System Diagram.

input (Yu and Salari,2011), (Vupparaboina et. al,2015) where different regions of the laser colours were extracted with the help of image processing, thermal imaging was also used by fusing thermal imaging and convolutional neural networks (Aparna et al.,2019) while others used visible light RGB cameras with supervised learning techniques by analysing the road's surface feature in order to classify potholes via HOG feature extraction (Azhar et al.,2016), while (Koch and Brilakis,2011) used segmentation in order to split mages into two categories (defective and non-defective) via the use of the histogram approach with shape-based thresholds [(Ryu et. al,2015), (Schiopu et. al,2016), and (Saluja et. al,2019) on the other hand used video sequences taken by RGB cameras via their own thresholding algorithm which considers potholes as the images with high-intensity values. Other techniques such as Probabilistic Generative Models (PGM) fused with Support Vector Machine (SVM) techniques were used in order to detect the probability of occurrence of a road crack via the intensity details was used by (Ai et. al,2018) while (Youquan et. al,2011), (Zhang et. al,2016) and (Li et. al,2018) have relied on stereo-vision techniques in different setups in order to detect potholes via their shapes, or by estimating the difference between the surface of the road and the surface of the pothole. (Moazzam et. al,2013) used a depth camera via the detection of the area, depth, length, width and volume of the pothole, while (Avellaneda and López-Parra,2016), (Buttlar and Islam,2014), and (Forslöf and Jones,2015) have used the accelerometer, compass and GPS found in mobile phones in order to achieve post-pothole detection, when (Chellaswamy et. al,2018) have used ultrasonic sensors. A more detailed review can be found in our previous publication *A Review on Negative Road Anomalies* (Dib et. al,2020)

The previously mentioned techniques were all limited due to the fact that post-detection of potholes cannot be used in order to avoid potholes. Ultrasonic sensors, laser-imaging techniques, surface difference-based techniques and depth camera-based techniques are limited when it comes to water-filled potholes as water can be reflective, and there will be no or insignificant surface difference between the surface of the water and the surface of the pothole.

This paper describes the current progress of the negative anomaly detection project and proposes the use of a normal RGB camera where the stream being fed to a custom-trained YOLO network which will achieve the real-time detection of the pothole. A dataset of pothole images was collected, preprocessed and used to train the neural network in order to fulfil the task required.

2 PROPOSED APPROACH

We propose the use of deep learning neural networks, mainly YOLO developed using the Darknet environment having an input obtained by an RGB camera mounted on any moving platform. This could be a self-driven car, driven car, truck, motorcycle, or even an electric-powered wheelchair or a robot.

A core computing unit will be mounted onto the moving platform. The RGB Camera's video feed will be processed by the computing unit which will be running the Robot Operating System (ROS) (ROS Wiki,2020) and will process the video feed and feed it into the pothole detection system (Figure 1).

First, the captured video feed will be pre-processed by converting the feed into RGB format (if it is not already in RGB format), then, the frames are downscaled to 416x416 pixels. Padding is used in the event where the downscaled frames have either a height or a width less than 416 pixels. Then, the frames will be processed by the YOLO convolutional neural network in order to detect and localise the potholes based on the features which the network is trained to detect. Potential pothole candidates will be detected, and the probability of the candidate being a true positive detection will be calculated according to the formulas discussed in Part 3 of this paper. If the

network's confidence is more than 0.7 (70%) the detected object is considered a pothole, it will be considered a positive detection, the x1,y1,x2,y2 coordinates of the bounding box are calculated, and the bounding box is drawn around it marking its location within the video frame. At this stage, this approach ensures reliability, scalability, and high performance.

Our approach's main goal is to ensure real-time detection with the least possible computing and power requirements. This will enable the use of the system in real-time scenarios without the need to rely on equipment with high computational power which could drain the battery used. This will ensure the ability to use our system in real-life scenarios and potentially provide a standard platform for both negative and positive obstacle detection and avoidance.

3 EXPERIMENT

For this experiment (Figure 2), different versions of the YOLO network were trained on a self-collected dataset which includes photos from different scenarios which could be encountered within an everyday usage of any vehicle/moving platform.

The images dataset which has been used to train the neural network was collected during the research phase using a Samsung Galaxy Note 8 phone camera taking images at 13 Megapixels. Images were collected within Kent County in the United Kingdom from different roads and cities in different weather conditions (sunny, cloudy, and rainy days and nights). Figure 4 presents a sample from our dataset:

(a) represents a general-looking pothole, i.e. a hole within the tarmac in an quasi-circular shape.

(b) represents a shallow circular broken surface/crack within the tarmac floor which is usually hard to detect by laser-based systems.

(c) represents a stochastic-shaped pothole, filled with water and dirt with a completely different pattern than the tarmac surrounding it. This cannot be detected by laser-based or sonar-based systems.

(d) represents a random-shaped pothole filled with water which is nearly clear and located on the side of the road where the double lines are clearly shown making it very difficult to be detected by normal image-based systems, laser-based systems, and sonar-based systems.

(e) represents a stochastic-shaped broken side of the road filled with rabble which is also very challenging for image-based, laser-based, and sonar-based systems due to the stochastic shape, and the reflective randomly-located rabble.

This dataset will be made available online at a later stage.

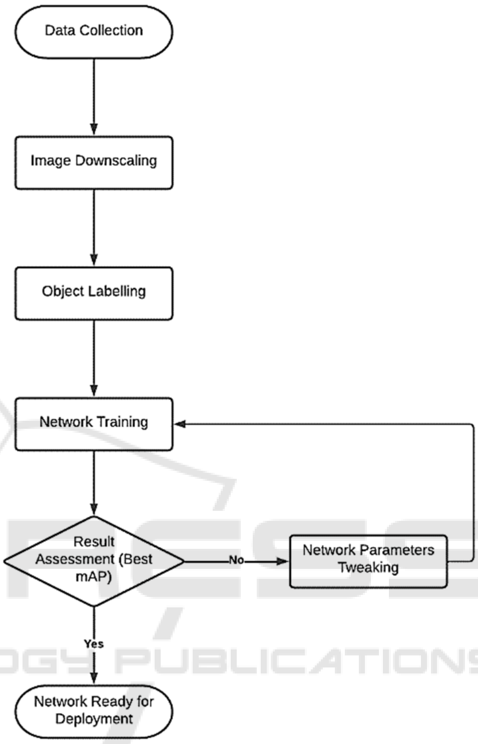

Figure 2: Network Training Process.

The collected images were pre-processed by downscaling them to 30% in order to obtain a width of around 415 pixels and then they were labelled individually using the labelling tool LabelImg (GitHub,2020) for Python ensuring that all the surrounding boxes cover the exact corners of the pothole without adding a lot of background data which could cause any diversion in the learning process.

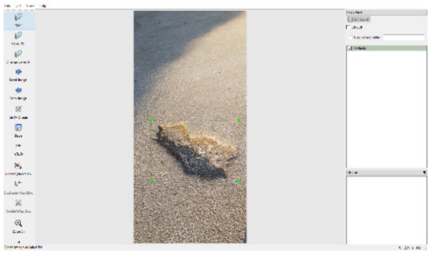

Figure 3: Labeling the dataset images via LabelImg.

The network training phase was done by using more than one platform and more than one version of YOLO in order to collect numerous test results and perform benchmarking.

All training experiments were done using an Intel Core i7 desktop with an NVIDIA RTX2080 6GB memory graphical processing unit (GPU) running a Windows 10 OS with Anaconda (Anaconda,2020) as a platform to run the Python environment on Windows.

In order to calculate the accuracy of the detection denoted by precision, we have considered the detection a true positive if the maximum overlap between the detected region (detected box) and the original annotation (annotated box of the RoI (Region of interest i.e. pothole) within the validation dataset) is larger than or equal to the Intersection over Union (IoU) (Rosebrock,2020) which is the area of overlap between the detected region and the annotation region divided by the area of the union which is the union of both areas combining the detected region and the annotated region:

After calculation, the precision of every detection, the mean average precision (mAP) is calculated via the calculation of the precision envelope, the area under the curve (points where the recall changes) and then the summing of those values. This has been described extensively in (Medium,2020)

The precision and recall formulas are as follows:

$$Precision = \frac{TP}{TP + FP}$$

$$Recall = \frac{TP}{TP + FN}$$

where TP is the number of true positives, FP is the number of false-positive, and FN is the number of false negatives.

The mean average precision (mAP) is the mean of the average precisions calculated, i.e. the sum of all average precisions divided by the number of detections.

$$mAP = \frac{\sum_{i=1}^{N} AP}{N}$$

where N is the number of detections, and AP is the Average Precision (described in (Anaconda,2020))

In addition to the previous values, we have recorded the frame rates achieved by the algorithm when tested in order to assess whether the detection is in real-time or not.

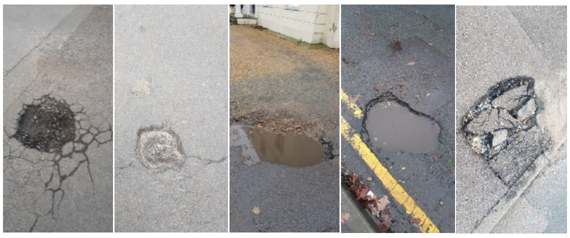

(a) (b) (c) (d) (e)

Figure 4: Samples images taken from our manually-collected dataset.

In our training experiments, we have set the IoU to 70 so detection is considered a true positive when there is an overlap that is more than 70% (YOLO uses a default IoU threshold of 0.3 (30%) which we have raised to 0.7).

We have attempted more than one training experiment and split them into three main sets. In every set, we have tried several different number of training and validation datasets as follows:

- 80% of the total number of images in the dataset is used as training data, the rest is used as validation images.
- 70% of the total number of images in the dataset is used as training data, the rest is used as validation images.
- 60% of the total number of images in the dataset is used as training data, the rest is used as validation images.

We have also attempted more than one different learning rate, as follows:

- Learning Rate = 1e-4
- Learning Rate = 1e-5
- Learning Rate = 9e-5

The first training set of experiments was made using YOLOv2 (Redmon et al., 2015) trained using Keras (Team K.,2020) (Keras Website) open-source neural network library with the following training parameters:

- Training Images: 574 (80% of the dataset)
- Validation Images: 143 (20% of the dataset)
- Learning Rate = 1e-4
- IoU = 0.7

The training resulted in a best mAP of 71.5%, 102 true positives, 41 false positives, and upon testing the neural network performance on a “challenging” input with more than one pothole present, the following results were obtained:

SCITEPRESS SCIENCE AND TECHNOLOGY PUBLICATIONS

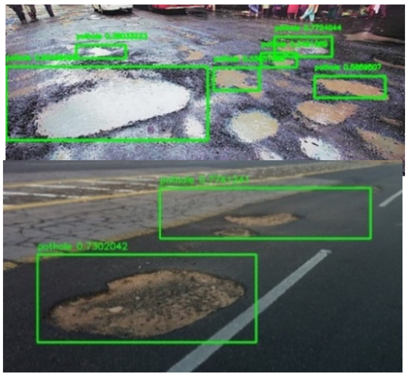

Figure 5: YOLOv2 Test Results.

It is evident from the first test results (Figure 5) that this network is not really fit for purpose as its result is a larger region of interest (ROI) detected versus the optimal ROI which should have been detected. A larger ROI can be acceptable as it means that the network has detected the object of interest which means that avoidance can be achieved, however, our aim is to obtain the most optimal detection possible. In addition, the network has detected only 6 potholes within the first example and only two in the second example along with a larger ROI where two potholes were considered as only one. Not to mention that the detection accuracy is only 0.7302 for the bottom pothole in the second example which is 73.02% for the most evident pothole. As for the frame rate achieved, it was 44 frames per second as reported by the algorithm.

The second training set of experiments was made using YOLOv3 (Redmon and Farhadi, 2018) trained using Keras with the same parameters as the previous training.

The network's best mAP was 75.48%, the number of true positives was 108 along with 35 false positives and the test input returned the following results:

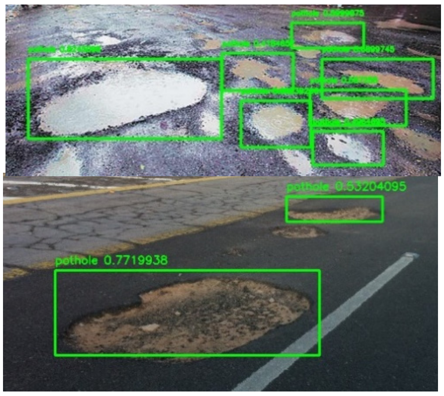

Figure 6: YOLOv3 Test Results.

The results obtained via YOLOv3 look slightly more promising than YOLOv2 as the network has detected 7 potholes in the first example and only two in the second but with tighter ROIs covering the exact borders of the potholes. In the second example, the evident pothole's detection accuracy was 0.7719 which is 77.19% which shows a slight improvement from the first network used. The frame rate was almost the same as the previous test (47 frames per second)

The training set of experiments was made using YOLOv4 (Bochkovskiy et. al, 2020) trained using Darknet environment with the same parameters as the previous training. The network's best mAP was 82.05%, the number of true positives was 117, and the false positives were 26. The test input returned the following results:

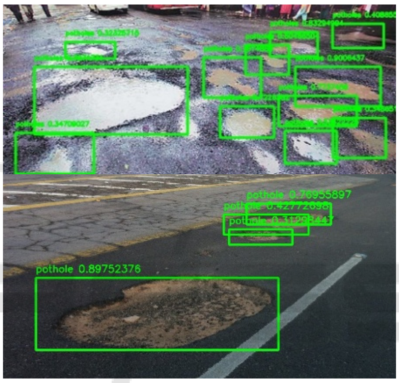

Figure 7: YOLOv4 Test Results.

The results obtained via YOLOv4 were the most promising results as the network has detected 12 potholes within the first example along with three potholes in the second example as the network has identified the two small potholes as an additional partial pothole. The accuracy of the detection for the evident pothole is 0.8975 which is 89.75% which was the highest obtained accuracy within the tests aforementioned. This experiment was achieved with a frame rate of 49 frames per second which is the highest frame rate achieved in all of our experiments.

Knowing that the speed limit for vehicles in residential areas in England is 30 mph (48.28 km/h or 13.411 m/s), and for other moving platforms such as electric-powered wheelchairs (EPWs) is 4 mph (6 km/h or 1.67 m/s) offroad (on a footpath, on a pavement, etc.) and 8mph (12 km/h or 3.3333 m/s) on the road (on tarmac) we can easily calculate our detection-rate via the formula:

$$Detection\ Rate = \frac{Frame\ Rate}{Max\ Speed}$$

SCIENCE AND TECHNOLOGY PUBLICATIONS

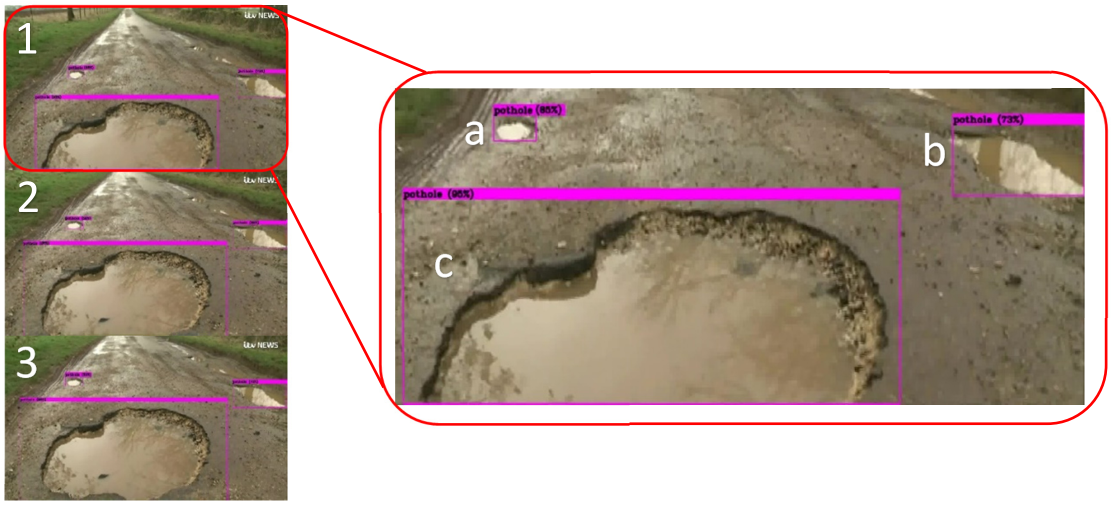

Figure 8: Real-time testing of the YOLO Network applied on an online video [29] where three consecutive frames have been extracted. In the test above, the precision rate of the detection is respectively: pothole 1.a: 85%, pothole 1.b: 73%, and pothole 1.c: 95% pothole 2.a: 56%, pothole 2.b: 82%, and pothole 2.c: 97%. pothole 3.a: 53%, pothole 3.b: 70%, and pothole 3.c: 96%. The Average Frame Rate achieved is 52 fps which is considered real-time.

By applying this formula to vehicles in rural areas, we can conclude that our detection rate is

$$\frac{49}{13.411} = 3.65$$

i.e. 3.65 frames per meter, as for the off-road wheelchair, it is 29.34 frames per meter and the on-road wheelchair detection rate is 14.70 frames per meter. These detection rates achieved are more than sufficient for safe navigation and avoidance of the pothole as the lowest detection rate (3.65 achieved when the system is mounted to cars in rural areas) allows at least 3 frames to be detected within every meter and as is known, 49 frames per second are larger than the commonly-used real-time threshold for frame-rate which is 30 frames per second. Figure 8 represents the real-time results obtained when using an mp4 video (ITV News YouTube Channel, 2018)as input to the network. In order to demonstrate the result, we have extracted three consecutive frames from the resulting video showcasing the detection rate achieved with the real-time frame rate of an average of 52 frames per second.

In addition to the previous training attempts, a separate attempt was made with the same training and validation ratios, along with different learning rates in order to attempt to find additional methods of improving the detection and studying the effect of the learning rate and the training/validation ratio on the mean average precision of the network's detection performance. The results were as follows:

Table 1: YOLO with Learning Rate = 1e-4.

Ratio Training/Validation	0.8	0.7	0.6
mAP	0.765	0.725	0.702
True Positives	109	156	302
False Positives	34	59	128

Table 2: YOLO with Learning Rate = 2e-4.

Ratio Training/Validation	0.8	0.7	0.6
mAP	0.5939	0.474	0.455
True Positives	85	102	196
False Positives	58	113	234

Table 3: YOLO with Learning Rate = 1e-5.

Ratio Training/Validation	0.8	0.7	0.6
mAP	0.521	0.497	0.434
True Positives	75	107	187
False Positives	68	108	243

Table 4: YOLO with Learning Rate = 9e-5.

Ratio Training/Validation	0.8	0.7	0.6
mAP	0.616	0.605	0.603
True Positives	88	130	259
False Positives	55	85	171

Table 5: YOLO with Learning Rate = 8e-5.

Ratio Training/Validation	0.8	0.7	0.6
mAP	0.681	0.671	0.532
True Positives	97	144	229
False Positives	46	71	201

Table 6: YOLO with Learning Rate = 7e-5.

Ratio Training/Validation	0.8	0.7	0.6
mAP	0.617	0.582	0.532
True Positives	88	125	229
False Positives	55	90	201

Table 7: YOLO with Learning Rate = 6e-5.

Ratio Training/Validation	0.8	0.7	0.6
mAP	0.615	0.647	0.543
True Positives	88	139	233
False Positives	55	76	197

As we can observe from this experiment, the detection's mAP is generally larger when the number of images used for training is larger which can be observed when comparing the mAP values at the different training/validation ratios. In addition, we can observe that setting the learning rate to 1e-4 returned the highest mAP value. However, 8e-5 and 9e-5 did return acceptable results at a training/validation ratio of 0.8 which leads us to conclude that a variable learning rate would be more ideal in respect to the validation loss, this could improve the results obtained and will be assessed in future work.

4 CONCLUSIONS

In this paper, we have implemented a deep learning-based system which detects and localises different types of potholes regardless of the stochasticity in their shapes, textures, patterns, and colours in real-time (i.e. high frame rates achieved within the experiments undertaken), and with high accuracy.

The results obtained show that the accuracy of the detection was very high even in the case of water-filled potholes which is usually considered the main limitation of many sensing techniques. Not to mention that the detection rate and the frame rates achieved were more than sufficient for our detection rate to be considered real-time providing sufficient detection speed and distance for a safe navigation and avoidance of potholes.

We can also conclude that the training results could be improved by varying the learning rate throughout the learning process, and by increasing the size of the training dataset used.

The next steps will focus on further use of deep learning object detection convolutional neural networks. In future work, there will focus on including more functionalities, such as object localization in real-world 3D coordinates and more additional functionalities via the use of additional sensing techniques fused by a data fusion algorithm. This algorithm will combine the use of more than one sensing technique in such a way that every technique used will cover the other techniques' weaknesses and limitations via the use of multimodal sensing techniques combined with deep learning. The proposed algorithm could be the backbone of a wide range of systems and it can be used to make decisions ensuring safe navigation of the moving platform when needed.

ACKNOWLEDGEMENTS

We would like to acknowledge the help Engineering and Physical Sciences Research Council (EPSRC) who funded the project via studentship reference 2112938 and Assistive Devices for empowering dis-Abled People through robotic Technologies (ADAPT). ADAPT is selected for funding by the INTERREG VA France (Channel) England Programme which is co-financed by the European Regional Development Fund (ERDF).

REFERENCES

J. Redmon, S. Divvala, R. Girshick. and A. Farhadi, 2015. IEEE Conference on Computer Vision and Pattern Recognition (CVPR). You Only Look Once: Unified, Real-Time Object Detection. [online] IEEE. Available at: <https://arxiv.org/abs/1506.02640> [Accessed 1 July 2020].

J. Redmon, 2020. Darknet: Open Source Neural Networks In C. [online] Pjreddie.com. Available at: <https://pjreddie.com/darknet/> [Accessed 1 July 2020].

X. Yu and E. Salari, 2011. ''Pavement pothole detection and severity measurement using laser imaging,'' in Proc. IEEE Int. Conf. Electro/Inf. Technol., Mankato, MN, USA, pp. 1–5.

Y. Aparna, R. Bhatia, V. Rai, N. Gupta, Aggarwal, and A. Akula, 2019. ''Convolutional neural networks based

potholes detection using thermal imaging,'' J. King Saud Univ.-Comput. Inf. Sci.,, doi: 10.1016/j.jksuci.2019.02.004.

K. Azhar, F. Murtaza, M. H. Yousaf, and H.A. Habib ,2016. ''Computer vision based detection and localization of potholes in asphalt pavement images,'' in Proc. IEEE Can. Conf. Electr. Comput. Eng. (CCECE), pp. 1–5.

C. Koch and I. Brilakis, 2011. ''Pothole detection in asphalt pavement images,'' Adv. Eng. Inform., vol. 25, no. 3, pp. 507–515.

S.-K. Ryu, T. Kim and Y.-R. Kim, ''Image based pothole detection system for ITS service and road management system, 2015.'' Math. Problems Eng.,vol. 2015, Art. no. 968361, doi: 10.1155/2015/968361.

I. Schiopu, J. P. Saarinen, L. Kettunen, and I. Tabus, 2016. ''Pothole detection and tracking in car video sequence,'' in Proc. 39th Int. Conf. Telecommun. Signal Process. (TSP), Vienna, Austria, pp. 701–706.

D. Ai, G. Jiang, L. Siew Kei, and C. Li, 2018. ''Automatic pixel level pavement crack detection using information of multi-scale neighborhoods,'' IEEE Access, vol. 6, pp. 24452–24463.

H. Youquan, W. Jian, Q. Hanxing, W. Zhang, and X. Jianfang, 2011. ''A research of pavement potholes detection based on three-dimensional projection transformation,'' in Proc. 4th Int. Congr. Image Signal Process., pp. 1805–1808.

L. Zhang, F. Yang, Y. Daniel Zhang, and Y. J. Zhu, 2016. ''Road crack detection using deep convolutional neural network,'' in Proc. IEEE Int. Conf. Image Process. (ICIP), pp. 3708–3712.

Y. Li, C. Papachristou, and D. Weyer, 2018. ''Road pothole detection system based on stereo vision,'' in Proc. IEEE Nat. Aerosp. Electron. Conf. (NAECON), pp. 292–297.

I. Moazzam, K. Kamal, S. Mathavan, S. Usman, and M. Rahman, 2013. ''Metrology and visualization of potholes using the microsoft kinect sensor,'' in Proc. 16th Int. IEEE Conf. Intell. Transp. Syst. (ITSC),pp. 1284–1291

D. A. Casas Avellaneda and J. F. López-Parra, 2016. ''Detection and localization of potholes in roadways using smartphones,'' DYNA, vol. 83, no. 195, pp. 156–162.

W. G. Buttlar and M. S. Islam, 2014. Integration of Smart-PhoneBased Pavement Roughness Data Collection Tool With Asset Management System—National Transportation Library. Accessed: May 20, 2019. [Online]. Available: https://rosap.ntl.bts.gov/view/dot/38287

Forslöf, L. and Jones, H., 2015. Roadroid: Continuous Road Condition Monitoring with Smart Phones. Journal of Civil Engineering and Architecture, 9(4).

C. Chellaswamy, H. Famitha, T. Anusuya, and S. B. Amirthavarshini, 2018.''IoT based humps and pothole detection on roads and information sharing,'' in Proc. Int. Conf. Comput. Power, Energy, Inf. Commun. (ICCPEIC), pp. 084–090.

Wiki.ros.org, 2020. ROS/Introduction - ROS Wiki. [online] Available at: <http://wiki.ros.org/ROS/Introduction> [Accessed 1 July 2020].

GitHub, 2020. Tzutalin/Labelimg. [online] Available at: <https://github.com/tzutalin/labelImg> [Accessed 1 July 2020].

Anaconda, 2020. Anaconda | The World's Most Popular Data Science Platform. [online] Available at: <https://www.anaconda.com/> [Accessed 1 July 2020].

Medium, 2020. Breaking Down Mean Average Precision (Map). [online] Available at: <https://towardsdatascience.com/breaking-down-mean-average-precision-map-ae462f623a52> [Accessed 1 July 2020].

A. Rosebrock, 2020. Intersection Over Union (Iou) For Object Detection - Pyimagesearch. [online] PyImageSearch. Available at: <https://www.pyimagesearch.com/2016/11/07/intersection-over-union-iou-for-object-detection/> [Accessed 1 July 2020].

Team, K., 2020. Keras: The Python Deep Learning API. [online] Keras.io. Available at: <https://keras.io/> [Accessed 1 July 2020].

J. Redmon, and A. Farhadi, 2018. Yolov3: An Incremental Improvement. [online] arXiv.org. Available at: <https://arxiv.org/abs/1804.02767> [Accessed 1 July 2020].

A. Bochkovskiy, C. Wang, and H. Liao, 2020. Yolov4: Optimal Speed And Accuracy Of Object Detection. [online] arXiv.org. Available at: <https://arxiv.org/abs/2004.10934> [Accessed 1 July 2020].

J. Dib, K. Sirlantzis and G. Howells, 2020. "A Review on Negative Road Anomaly Detection Methods," in IEEE Access, vol. 8, pp. 57298-57316, 2020, doi: 10.1109/ACCESS.2020.2982220.

Vupparaboina, K.K., Tamboli, R.R., Shenu, P.M., & Jana, S., 2015. Laser-based detection and depth estimation of dry and water-filled potholes: A geometric approach. 2015 Twenty First National Conference on Communications (NCC), 1-6.

I. Saluja, R. Karwa, A. Bharambe, M Sabnis, 2019."Recognition and Depth Approximation of Dry & Filled Potholes", International Journal of Innovative Research in Science, Engineering and Technology, Vol. 8, Issue 8.

ITV News YouTube Channel,11 May 2018. Potholes costing drivers and insurers '£1m a month' | ITV News. Available at: <https://www.youtube.com/watch?v=MlDI-zlcc8I> [Accessed 15 September 2022].
